# Splenic Diffuse Red Pulp Small B-Cell Lymphoma with Overlapping Clinical and Immunophenotypic Features with Hairy Cell Leukaemia: A Case Report and a Review of the Literature

**DOI:** 10.3390/genes16040467

**Published:** 2025-04-19

**Authors:** Mirette Hanna, Michola Trus, Erica DiMaria

**Affiliations:** 1 Department of Pathology and Laboratory Medicine, Royal Victoria Regional Health Center, Barrie, ON L4M 6M2, Canada; 2 Department of Laboratory Medicine and Pathobiology, University of Toronto, Toronto, ON M5S 1A8, Canada; 3 Department of Pathology and Molecular Medicine, McMaster University, Hamilton, ON L8S 4K1, Canada; 4 Division of Hematology, Department of Oncology, Royal Victoria Regional Health Center, Barrie, ON L4M 6M2, Canada

**Keywords:** splenic B-cell lymphomas and leukaemias, splenic diffuse red pulp small B-cell lymphoma, hairy cell leukaemia, splenic B-cell lymphoma/leukaemia with prominent nucleoli, splenic marginal zone lymphoma

## Abstract

Background: Splenic B-cell lymphomas and leukaemias include hairy cell leukaemia, splenic marginal zone lymphoma, splenic diffuse red pulp small B-cell lymphoma, and splenic B-cell lymphoma/leukaemia with prominent nucleoli. The main diagnostic challenge is to differentiate between splenic B-cell lymphomas and leukaemias due to highly overlapping clinical, morphologic, and phenotypic features in the absence of splenectomies for diagnostic purposes. Case presentation: We describe a case of a 78-year-old woman who presented with weight loss and was subsequently found to have pancytopenia, lymphocytosis, and splenomegaly. Peripheral blood smear showed a homogenous population of small- to medium-sized lymphocytes having oval nuclei, condensed chromatin, and villous cytoplasmic projections. Bone marrow showed B-cell infiltrate in a predominantly intrasinusoidal pattern. Except for cyclin D1 and BRAF, the immunophenotype was similar to that of hairy cell leukaemia. This was further supported by the lack of *BRAF* p.V600E mutation by polymerase chain reaction. A diagnosis of splenic diffuse red pulp small B-cell lymphoma was thus favored based on the lack of cyclin D1 expression and pattern of infiltration in the bone marrow biopsy. Conclusions: Awareness of this infrequent clinical presentation and immunophenotype of splenic diffuse red pulp small B-cell lymphoma is crucial for diagnosis and devising appropriate therapeutic strategies for the patient.

## 1. Introduction

Splenic B-cell lymphomas and leukaemias are rare lymphoid malignancies that include hairy cell leukaemia (HCL), splenic marginal zone lymphoma (SMZL), splenic diffuse red pulp small B-cell lymphoma (SDRPL), and splenic B-cell lymphoma/leukaemia with prominent nucleoli (SBLPN) [1]. While splenic B-cell lymphomas and leukaemias have a peculiar tendency to present in the spleen, bone marrow and peripheral blood are involved in most cases. They share overlapping clinical, cytomorphologic, and immunophenotypic features, posing difficulties in the reliable distinction between the splenic B-cell lymphomas and leukaemias, except for HCL harboring the characteristic *BRAF* p.V600E mutation.

In this report, we describe a 78-year-old woman having splenic B-cell lymphoma favoring SDRPL with overlapping clinical and immunophenotypic features with HCL. SDRPL was previously thought to be a variant of SMZL with a diffuse pattern of splenic involvement [2]. In the 2008 WHO Classification of Tumours of Haematopoietic and Lymphoid Tissues, it was recognized as a provisional separate entity. Currently, SDRPL is classified separately in the splenic B-cell lymphomas and leukaemias in the 5th edition of the WHO Classification of Haematolymphoid Tumors [3]. SDRPL accounts for <1% of all lymphoid malignancies with peripheral blood involvement [3]. It has a slight male predominance, with a median age at presentation of 65 years [4,5]. Almost all patients present with splenomegaly and low-level lymphocytosis, while pancytopenia and B-symptoms are infrequent. Splenic biopsy shows diffuse and intrasinusoidal infiltration by monotonous small lymphocytes associated with white pulp atrophy. The peripheral blood smear shows a predominance of villous lymphocytes having unevenly distributed small cytoplasmic projections, round to oval nuclei, condensed chromatin and absent or indistinct nucleoli. SDRPL almost always infiltrates the bone marrow in a predominantly intrasinusoidal pattern. Immunophenotypically, neoplastic cells show characteristic expression of cyclin D3, in addition to DBA44, surface immunoglobulins, and mature B-cell phenotype [3,6]. Molecular data on SDRPL are limited. Notwithstanding, recurrent mutations have been reported in *BCOR*, *CCND3*, and genes involved in the NOTCH pathway, including *MAP2K1* [7]. Our understanding of SDRPL has advanced considerably. However, multiple inconsistencies remain regarding diagnostic criteria.

This case highlights the difficulties in the reliable distinction between splenic B-cell lymphomas and leukaemias due to considerable overlap in histology and immunophenotyping. A review of the literature, including key distinguishing points, is provided.

## 2. Detailed Case Description

A 78-year-old woman presented to her primary care physician for unintentional 20 pounds of weight loss over 6 months. She denied any fevers or drenching night sweats. Initial work-up revealed normocytic anemia; hemoglobin of 11.8 g/dL (reference range (RR): 12.0–16.0 g/dL); thrombocytopenia, 105 × 10^9^/L (RR: 150–400 × 10^9^/L); lymphocytosis, 6.2 × 10^9^/L (RR: 1.0–3.5 × 10^9^/L); WBC and absolute neutrophil count (ANC) within normal limits, 10.2 × 10^9^/L and 3.3 × 10^9^/L, respectively (RR: 4.0–11.0 and 2.0–7.5 × 10^9^/L, for WBC and ANC, respectively) and ultrasound-confirmed splenomegaly, measuring 19 cm. Her past medical history was significant for irritable bowel syndrome (IBS) and obstructive sleep apnea. 

**Figure 1:**
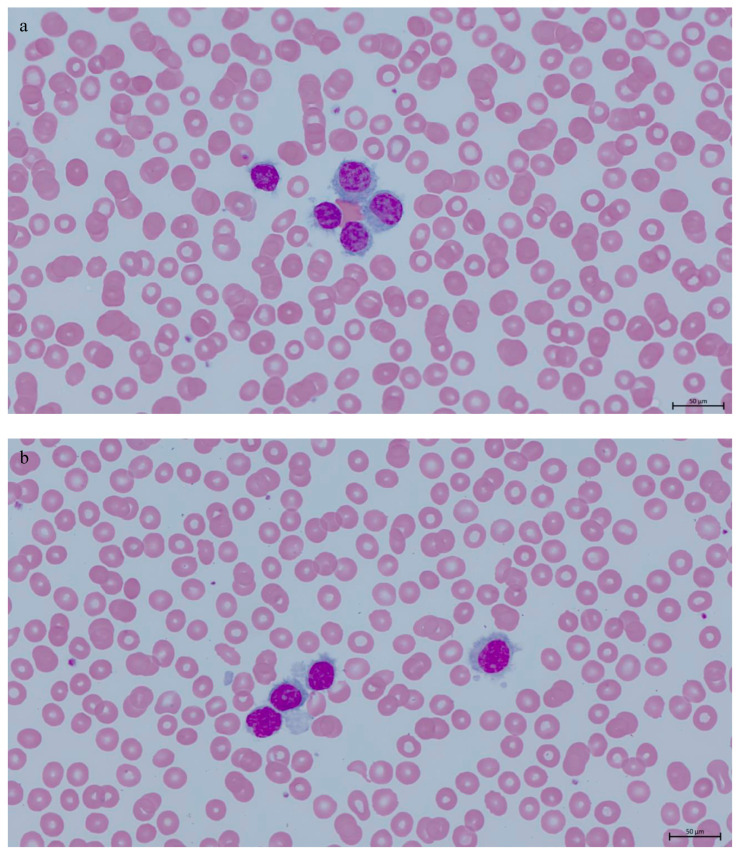
(**a**,**b**) Peripheral blood smear with Wright–Giemsa staining showing atypical small- to medium-sized lymphoid cells having oval nuclei, condensed chromatin, variable amount of basophilic cytoplasm with unevenly distributed projections and no prominent nucleoli, ×63 oil immersion.

The patient was subsequently referred to the hematology clinic at the Royal Victoria Regional Health Center. At presentation in our institution, physical examination and computed tomography (CT) revealed no neck, thoracic or abdominopelvic lymphadenopathy. Laboratory work-up showed pancytopenia, hemoglobin of 10.7 g/dL (RR: 11.5–16.0 g/dL), MCV of 94.5 fL (RR: 80–95 fL), platelets of 112 × 10^9^/L (RR: 150–400 × 10^9^/L), WBC of 6.43 × 10^9^/L (RR: 4.0–11.0 × 10^9^/L), ANC of 0.98 × 109/L (RR: 2.00–7.50 × 10^9^/L), monocytes of 0.26 × 10^9^/L (RR: 0.00–1.20 × 10^9^/L), and lymphocytes of 5.00 × 10^9^/L (RR: 1.00–3.50 × 10^9^/L). Peripheral blood smear examination showed circulating small- to medium-sized lymphocytes having oval nuclei, condensed chromatin, variable amounts of basophilic cytoplasm with villous projections, and no prominent nucleoli (Figure 1). Peripheral blood flow cytometric analysis performed at Hamilton Regional Laboratory Medicine Program revealed a kappa-restricted B-cell population comprising approximately 30% of the total leukocytes and expressing bright CD19+, bright CD20+, CD5−, CD10−, bright FMC7+, partial dim CD23+, bright CD79b+, CD103+, CD11c+, CD25+, and CD123+. No paraprotein was detected by serum protein electrophoresis. A splenic biopsy was not performed.

**Figure 2:**
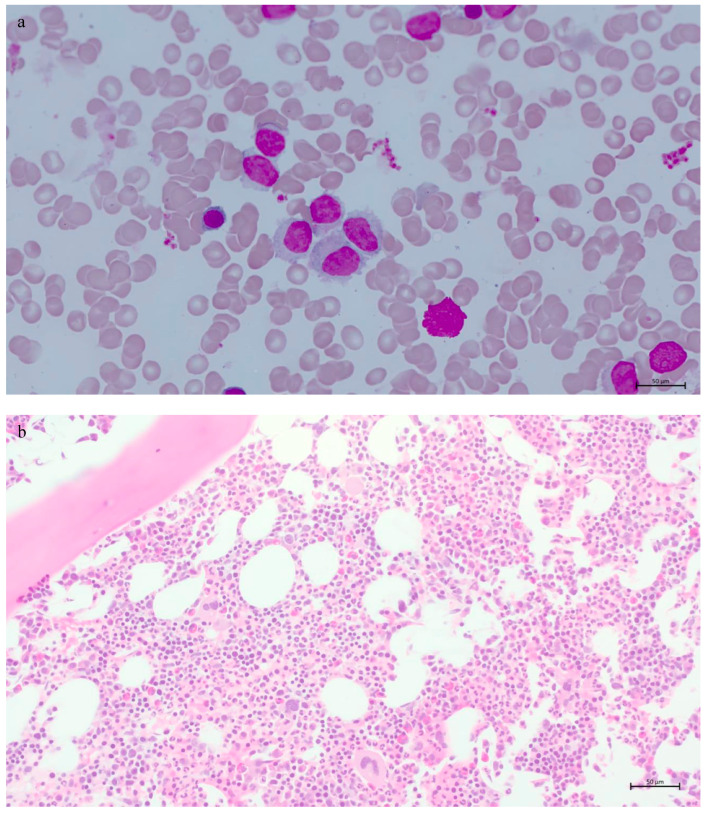
Histologic examination of the bone marrow aspirate and trephine biopsy. The bone marrow aspirate smear, showing small- to medium-sized lymphocytes having oval nuclei and villous cytoplasmic projections, ×63 oil immersion (**a**). Haematoxylin-eosin-stained bone marrow trephine biopsy showing no evident “fried-egg” appearance and diffuse increase in small- to medium-sized lymphocytes having moderate amount of cytoplasm, ×20 (**b**).

**Figure 3:**
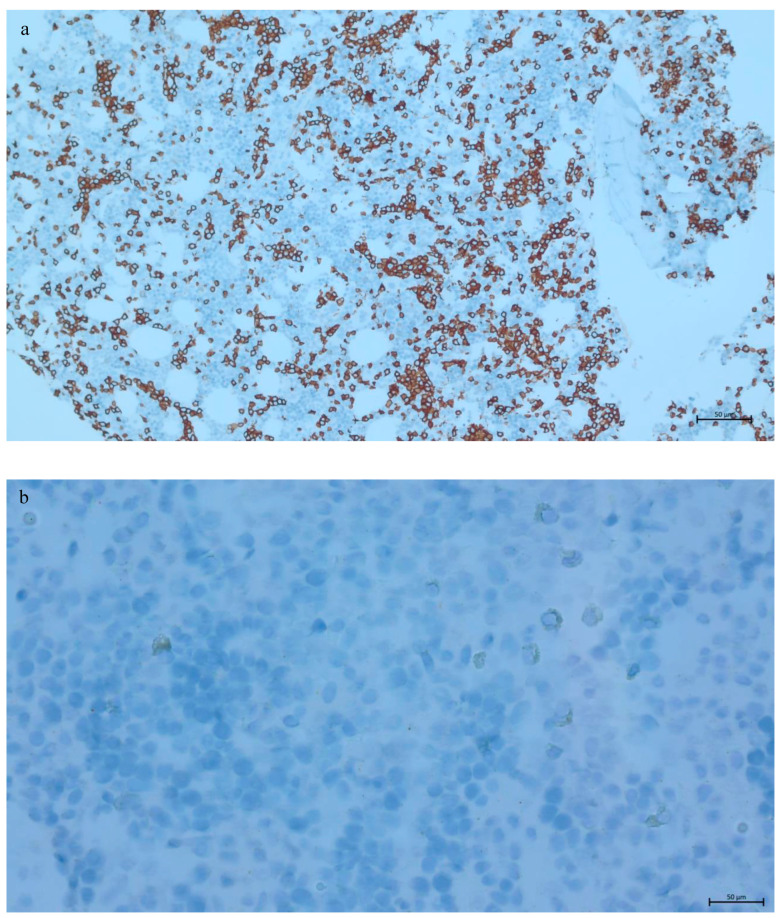

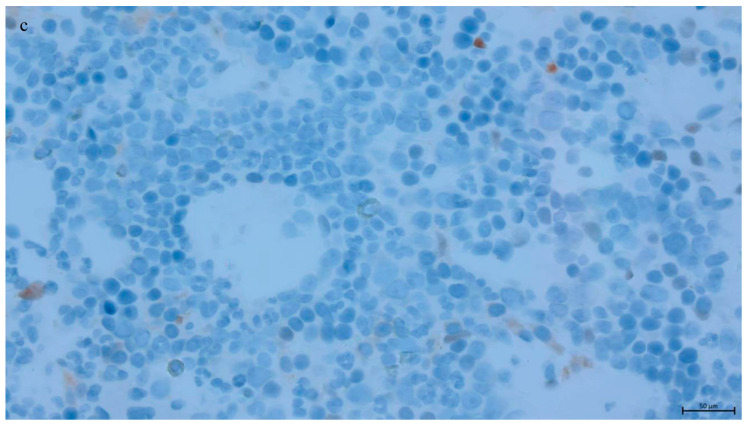
Immunophenotyping of B-cell lymphoid infiltrate in intrasinusoidal pattern highlighted by CD20, ×10 (**a**). The neoplastic cells are negative for BRAF (**b**) and cyclin D1 (**c**), ×40.

**Figure 4:**
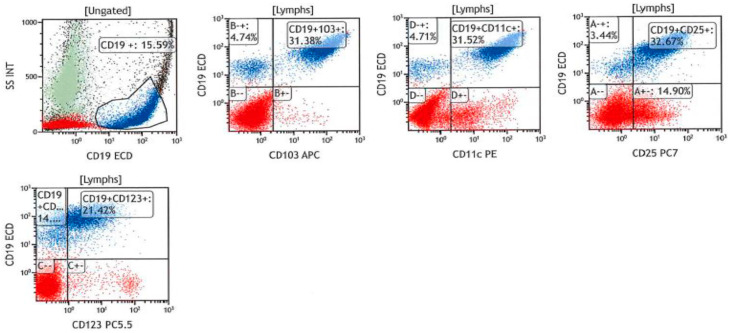
Bone marrow flow cytometry dot-plots showing neoplastic B-cell population (blue) expressing CD19, CD103, CD11c, CD25 and CD123.

Bone marrow biopsy revealed normal cellularity for age (30%) with increased lymphocytes (41% of the marrow cells by manual differential count), sufficient residual hematopoiesis, and mild reticulin fibrosis (grade 1/3) (Figure 2). The “fried-egg” appearance, typically seen in hairy cell leukaemia (HCL), was not evident on low-power examination. Neoplastic B-cells showed a predominantly intrasinusoidal and interstitial pattern of infiltration by CD20 immunohistochemical (IHC) staining while they were negative for CD5, CD10, cyclin D1, and BRAF (Roche 760–2531, 790–4451, 790–4506, 790–4508, and 760–5095 for CD20, CD5, CD10, cyclin D1, and BRAF, respectively) (Figure 3). IHC staining for DBA44 and ANXA1 was not available in our laboratory. Concurrent flow cytometric analysis of the bone marrow aspirate performed at Hamilton Regional Laboratory Medicine Program revealed a kappa-restricted B-cell population, comprising approximately 30% of the total leukocytes, and expressing bright CD19+, bright CD20+, CD5−, CD10−, CD23+, bright CD79b+, CD103+, CD11c+, CD25+, and CD123+ (Figure 4). The *BRAF* p.V600E mutation was not detected in the bone marrow sample by real-time PCR assay (EntroGen BRAF Codon 600 Mutation Analysis Kit). Chromosomal analysis by conventional karyotype was unremarkable except for a loss of a chromosome X detected in 3 out of 20 metaphases analyzed, which is suspected to be an artifact (45,X,-X [3]/46,XX [17]).

**Figure 5:**
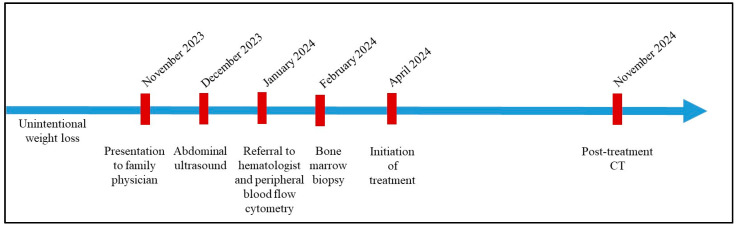
Patient’s clinical course timeline.

A diagnosis of bone marrow involvement by a splenic B-cell lymphoma and leukaemia was, therefore, made. In view of the morphologic and immunophenotype findings, the differential diagnosis included BRAF-negative HCL, SDRPL, and SMZL. The neoplastic cell morphology in the peripheral blood and immunophenotype, particularly expression of CD103 in addition to CD11c and CD123, made SMZL less likely. Likewise, SBPLN was considered to be unlikely, given the absence of large prominent nucleoli and the expression of CD25 on B-cells. SDRPL was favored over BRAF-negative HCL, based on the lack of cyclin D1 expression and pattern of infiltration in the bone marrow biopsy. Subsequently, the patient was treated with bendamustine and rituximab because of the cytopenia. The patient completed six cycles of treatment, and bendamustine was dose-reduced because of frailty. Post-treatment CT demonstrated resolution of the splenomegaly. The patient’s clinical course timeline is presented in Figure 5.

## 3. Discussion

We report a complicated case of a splenic B-cell lymphoma and leukaemia favoring SDRPL based on peripheral blood smear and bone marrow biopsy examination having high overlapping clinical and immunophenotypic features of HCL.

According to the most recent WHO haematolymphoid tumour classification, splenic B-cell lymphomas and leukaemias include HCL, SMZL, SDRPL, and SBLPN [1]. Splenic B-cell lymphomas and leukaemias are rare lymphoid malignancies, each accounting for <1–2% of all non-Hodgkin lymphomas [3,8,9,10]. They share some clinical, cytomorphologic, and immunophenotypic features posing difficulties in diagnosis. In contrast to HCL, the SMZL, SDRPL, and SBLPN have less defined immunophenotypic and genetic characteristics.

Clinically, patients with splenic B-cell lymphomas and leukaemias often present with splenomegaly and bone marrow and peripheral blood involvement with variable degrees of lymphocytosis [11]. However, pancytopenia with monocytopenia, commonly described in HCL, is infrequent in SDRPL and SBLPN [3,8,10]. Likewise, liver and nodal involvement other than splenic hilar nodes are infrequent in splenic B-cell lymphomas and leukaemias [3,8,9,10]. In fact, the absence of lymphadenopathy other than the splenic hilar lymph node is one of the desirable diagnostic criteria of SDRPL [3]. Autoimmune manifestations, present in approximately 20% of patients with SMZL, are rare in HCL and SBLPN [8,9,10]. Systemic B symptoms are infrequent in SMZL and SDRPL [3,9]. Our patient had no lymphadenopathy; however, contrary to the majority of reported SDRPL cases, she presented with pancytopenia and had significant weight loss over the 6 months preceding presentation. Similar to our patient, Kanellis et al. reported B symptoms in 35% of patients diagnosed with SDRPL in a 17-patient case series [12].

Morphologically, it is often challenging to distinguish these entities. Spleen biopsy is the gold standard for diagnosis; however, it is rarely performed. Diagnosis is often based on peripheral blood smear and bone marrow biopsy findings. Distinguishing morphologic findings include a pattern of infiltration in the spleen and bone marrow, the presence and distribution of villous cytoplasmic projections around neoplastic cells, the presence of prominent nucleolus, and the degree of bone marrow fibrosis. SMZL is differentiated from other splenic B-cell lymphomas and leukaemias by its nodular involvement of splenic white pulp with a targetoid appearance in addition to red pulp involvement [9]. HCL, SDRPL, and SBLPN typically diffusely involve the splenic red pulp without nodularity and with atrophic white pulp. Cytologic characteristics of the neoplastic lymphoid cells are best appreciated in the peripheral blood smears. In the peripheral blood, neoplastic lymphoid cells are small- to medium-sized with villous circumferential projections in HCL or unevenly distributed projections around the cells with a polar concentration in SMZL and SDRPL [11]. Nucleoli are inconspicuous or absent in HCL, SMZL, and SDRPL, while neoplastic cells in SBLPN have large, prominent single nucleolus reminiscent of prolymphocytes without circumferential cytoplasmic projections [11]. In the bone marrow biopsy, neoplastic cells in HCL exhibit a patchy or interstitial distribution with a characteristic “fried-egg” appearance, typically seen on low-power examination, attributable to the abundant cytoplasm widely spacing the nuclei and prominent cell-to-cell borders [13]. Neoplastic cells are distributed in intrasinusoidal and interstitial patterns in SMZL, SDRPL, and SBLPN, with a predominance of the intrasinusoidal pattern in SDRPL [11]. Nodular bone marrow infiltrates frequently seen in SMZL are rare in SDRPL [1]. In fact, predominantly nodular patterns or mixed intrasinusoidal/interstitial patterns were the most commonly reported infiltration patterns in SMZL in a paired assessment study, including 46 cases of SMZL and SDRPL [14]. In this study, Ponzoni et al. endorsed that pure patterns tended to be more frequent in SDRPL compared to SMZL [14]. Paired assessment of the spleen and bone marrow biopsy was a major asset in this study. Contrary to HCL that often presents with extensive reticulin fibrosis resulting in difficult bone marrow aspiration, SDRPL, and SBLPN exhibit mild or insignificant reticulin fibrosis [11]. Taken together, the architectural pattern of infiltration, as well as the presence of mild reticulin fibrosis observed in our case, were in favor of SDRPL over BRAF-negative HCL or SMZL.

Neoplastic B-cells detected in our patient showed aberrant expression of CD11c, CD25, CD103, and CD123 by flow cytometry and were negative for cyclin D1 by IHC. HCL typically expresses CD11c, CD25, CD103, CD123, ANXA1, cyclin D1, TRAP, and DBA44 [13,15]. SBLPN shares with HCL the expression of DBA44, CD11c, and CD103 but is often negative for CD25, CD123, ANXA1, cyclin D1, and TRAP [8]. Likewise, the expression of DBA44 in SDRPL was detected in most of the reported cases [5,12,16,17,18,19] with few exceptions [5,12,20], while ANXA1 and cyclin D1 are typically absent in SMZL and SDRPL. Expression of CD11c and CD123 has been reported in a subset of SMZL and SDRPL cases [5,12,17,21,22,23,24,25,26]. Nonetheless, expression of CD25 in SDRPL was reported in the spleen section of one case in a case series of 19 patients diagnosed with SDRPL, while none of the patients had CD25 expression in the peripheral blood [5]. In contrast, Kanellis et al. reported CD25 expression on circulating B-cells in 1 out of 10 cases of SDRPL included in their 17-patient case series, while they did not comment on CD25 expression in the spleen [12]. Interestingly, the two studies based their diagnosis on spleen examination and had a comparable study population (male predominance with a median age of 65 years). Although the immunophenotype of neoplastic cells in our patient was very similar to HCL, we thought that the lack of both cyclin D1 and BRAF was in favor of SDRPL over HCL. Neoplastic cells in our patient expressed CD103, which has been occasionally reported in a few SDRPL cases [6,12,17,21], while it is typically negative in SMZL [22,25,27,28], with very rare exceptions [29,30]. At this point, it should be noted that Ocio et al. reported dim CD103 positivity detected by flow cytometry in seven cases of SMZL [29], and this finding was not further replicated by IHC [27,31]. Yet, it remains to say that SMZL and SDRPL share, to a large extent, immunophenotypical features, precluding a definite diagnosis in the absence of a splenectomy specimen. On the other hand, cyclin D3 seems a promising marker in distinguishing SDRPL from other splenic B-cell lymphomas and leukaemias as it is reported to be exclusively expressed in approximately 70% of SDRPLs [3]. Unfortunately, this marker is not readily available in most laboratories.

Except for *BRAF* p.V600E, the genetic hallmark of HCL, no genetic mutation or chromosomal abnormality is pathognomonic for other splenic B-cell lymphomas and leukaemias. Albeit rare, BRAF wild-type HCL cases have been described. Approximately 40% of SBLPN cases harbour *MAP2K1* mutations, while these are infrequent in SMZL and SDRPL and absent in HCL [32]. Furthermore, the absence of *BRAF* p.V600E, *MYD88*, *NOTCH2*, and *TP53* mutation was reported in the majority of cases with SDRPL in a large case series [33]. Interestingly, *BRAF* p.V600E mutation was reported in 1 out of 42 SDRPL cases in a whole-exome sequencing study [34], and *MYD88* L265P and *NOTCH2* mutations were detected in one case each, while most cases displayed a mutated *IGHV* status [33]. Notwithstanding, *MYD88* mutated cases must be interpreted with caution, and the possibility of a lymphoplasmacytic lymphoma should be ruled out. Reported *BRAF*-positive SDRPL expressed CD103 while it lacked CD25 and CD123 expression [34]. Nonetheless, mutations in cyclin D3 (*CCND3*) or *BCOR* were identified in approximately one-quarter of patients with SDRPL, while these were rarely observed in HCL or SMZL [34,35]. Mutations in cyclin D3 (*CCND3*) were reported in approximately 25% of SBLPN [32]. Although uncommon, alterations of chromosome 7q were reported in occasional cases of HCL, SMZL, SDRPL, and SBLPN, suggesting a common pathogenic pathway [32,33,36]. Interestingly, the chromosome 7q alterations were not correlated with the other characteristic features of splenic B-cell lymphomas and leukaemias. Nonetheless, deletion of chromosomes 11q and 14q were detected in sporadic SDRPL cases [33].

SDRPL usually follows an indolent course with a five-year survival rate of >90%. So far, there are no established standard treatment strategies for SDRPL [12]. Treatment strategies commonly include watchful waiting, splenectomy, or rituximab monotherapy [12]. Limited data are available regarding the response to treatment. Moreover, very few studies described rare cases of transformation to aggressive lymphoma [6,12]. Kanellis et al. reported 4 out 17 patients experienced systemic and extra-nodal dissemination of the disease, with 1 of these patients diagnosed with large cell transformation [12]. Unfortunately, the histologic characteristics of transformation were not described. In addition, a sequencing study was not obtained at progression in Kanellis et al.’s case series [12]. Although factors associated with transformation are not fully established, it was suggested that the detection of a higher number of large-sized B-cells in the peripheral blood or bone marrow may predict an ongoing transformation process [32]. Moreover, the presence of mutations in *NOTCH1*, *TP53*, *MAP2K1, ARID2, CREBBP*, and *TNFRSF14* seems to be associated with aggressive disease and shorter progression-free survival [5,37]. Similar to SDRPL, progression to aggressive lymphoma was reported in a few HCL and SMZL cases [32]. SBLPN seems to follow a more aggressive course [38].

## 4. Conclusions

In summary, we have presented a patient with SDRPL having clinical and immunophenotypic features very similar to HCL except for the lack of expression of cyclin D1 and a marrow infiltration pattern favoring SDRPL. The awareness of such presentation is essential to clearly differentiate SDRPL from other splenic B-cell lymphomas and leukaemias. Considering the lack of spleen analysis in our case and that we relied on cytologic and immunophenotypic findings from peripheral blood and bone marrow, it will be important to see these data confirmed in additional cases. In addition, the lack of mutational analysis other than *BRAF* p.V600E provides another limitation of this study. Accordingly, a large cohort is required to establish the distinctive clinicopathological and molecular characteristics and may help elucidate treatment protocols.

## Data Availability

The data presented in this study are available upon request from the corresponding author.

## Abbreviations

ANC	absolute neutrophil count
CT	computed tomography
HCL	hairy cell leukaemia
IBS	irritable bowel syndrome
PCR	polymerase chain reaction
RR	reference range
SBPLN	splenic B-cell lymphoma/leukaemia with prominent nucleoli
SDRPL	splenic diffuse red pulp small B-cell lymphoma
SMZL	splenic marginal zone lymphoma
WBC	white blood cell
